# Supplementary material for: Polyketide synthase gene clusters in the ericoid mycorrhizal fungus Oidiodendron maius: genome-wide prediction, expression profiling, and functional characterization of knockout mutants
Source: Front Plant Sci. 2026 Sep 16;17:1924430. doi: 10.3389/fpls.2026.1924430 (PMC13623903; doi:10.3389/fpls.2026.1924430)
Supplement: Supplementary file 1 [file SupplementaryFile1.zip › Supplementary Materials/Supplementary Folder 1.zip!/Oidma1_AssemblyScaffolds_Repeatmasked/knownclusterblast/cluster45/jgi.p_Oidma1_106704_mibig_hits.html]

| MIBiG Protein | Description | MIBiG Cluster | MiBiG Product | % ID | % Coverage | BLAST Score | E-value |
| --- | --- | --- | --- | --- | --- | --- | --- |
| AGO59039.1 | PtaJ | BGC0000121 | Pestheic\_acid\_biosynthetic\_gene\_cluster | 36 | 99.1011235955 | 273 | 4e-73 |
| ACH72894.1 | AflY | BGC0000011 | Aflatoxin/sterigmatocystin\_biosynthetic\_gene\_cluster | 39 | 90.1123595506 | 272 | 8.9e-73 |
| AAS90068.1 | HypA | BGC0000009 | Aflatoxin\_biosynthetic\_gene\_cluster | 37 | 95.2808988764 | 260 | 3.5e-69 |
| CBF90088.1 | conserved\_hypothetical\_protein | BGC0001271 | Emericellin\_biosynthetic\_gene\_cluster | 36 | 100.674157303 | 254 | 1.9e-67 |
| AAS90113.1 | hypothetical\_protein | BGC0000006 | Aflatoxin\_biosynthetic\_gene\_cluster | 37 | 95.2808988764 | 254 | 2.5e-67 |
| AAS90089.1 | HypA | BGC0000010 | Aflatoxin\_biosynthetic\_gene\_cluster | 37 | 95.2808988764 | 254 | 2.5e-67 |
| AAS90039.1 | HypA | BGC0000008 | Aflatoxin\_biosynthetic\_gene\_cluster | 37 | 95.2808988764 | 253 | 3.3e-67 |
| BAE71335.1 | hypothetical\_protein | BGC0000004 | Aflatoxin\_biosynthetic\_gene\_cluster | 36 | 95.2808988764 | 249 | 8e-66 |
| EAL89345.1 | conserved\_hypothetical\_protein | BGC0001403 | Trypacidin\_biosynthetic\_gene\_cluster | 33 | 100.0 | 243 | 5.8e-64 |
| AAS90020.1 | HypA | BGC0000007 | Aflatoxin\_biosynthetic\_gene\_cluster | 35 | 95.2808988764 | 242 | 1.3e-63 |
| ADM34146.1 | hypothetical\_protein | BGC0001084 | Notoamide\_/\_stephacidin\_biosynthetic\_gene\_cluster | 32 | 101.573033708 | 210 | 3.2e-54 |
